# Reproducibility of strain and twist measurements calculated using CSPAMM tagging

**DOI:** 10.1186/1532-429X-13-S1-P52

**Published:** 2011-02-02

**Authors:** Peter Swoboda, Abdulghani Larghat, John Greenwood, Sven Plein

**Affiliations:** 1 Leeds General Infirmary, Leeds, UK; 2 University of Leeds, Leeds, UK

## Objective

To establish inter-observer and inter-study variability of CSPAMM (complementary spatial modulation of magnetization) tagged CMR data.

## Background

The reproducibility of CSPAMM-derived myocardial strain and left ventricular twist have not been described in detail.

## Methods

12 healthy volunteers (6 males, mean age 33±7 years) underwent CMR studies on a 1.5T system (Intera CV, Philips Healthcare, The Netherlands). Tagged CMR images were acquired at the apex, mid-ventricle and base with a CSPAMM pulse sequence (field of view: 300mm, matrix 128 x 128, slice thickness 10mm, tag separation 8mm, 18 phases, typical TR/TE 30ms/6ms, flip angle 25 degrees). In 6 volunteers repeat data sets were acquired after a mean interval of 8±3 days.

Data were analysed by two independent observers using Tagtrack software (Gyrotools, CH). Circumferential Langranian strain, radial Langranian strain and rotation were calculated for the three short axis slices. Endocardial and epicardial borders were drawn, and a midline calculated automatically. Left ventricular twist was calculated by subtracting the basal rotation from the apical rotation. The mean difference between paired measurements, the standard deviation (SD) of the differences and the coefficient of variability (Co-V) were calculated.

## Results

**Figure 1:**
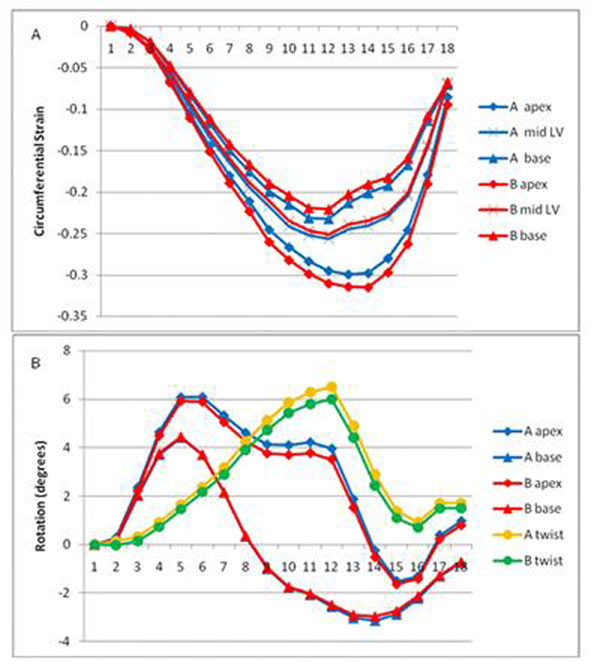
Figure 1a shows circumferential strain measured by observers A & B. Figure 1b shows rotation measured by observers A & B and calculated left ventricular twist

Circumferential strain increased from base to apex (Figure 1a). Mean (SD) difference in circumferential strain between the two operators was 6.9% (2.3), 8.2% (7.7) and 6.7% (5.0) at apex, mid-LV and base respectively. There was no significant difference in inter-observer variability in endocardial, midline and epicardial contours. See Table 1 for Co-V results.

**Table 1:** shows percentage difference and coefficient of variation (Co-V) for inter-observer and inter-study variability of measured circumferential strain, radial strain and left ventricular twist

		INTER-OBSERVER VARIABILITY		INTER-STUDY VARIABILITY	
	
				Percentage difference (mean (SD))	Co-V %	Percentage difference (mean (SD))	Co-V %
circumferential strain	apex	6.9 (2.3)	4.4	6.7 (4.1)	8.3
circumferential strain	mid LV	8.2 (7.7)	11.2	6.5 (4.0)	7.7
circumferential strain	base	6.7 (5.0)	6.8	8.8 (5.3)	10.8
radial strain	apex	28.1 (47.8)	19.2	7.3 (6.0)	9.0
radial strain	mid LV	17.6 (21.6)	18.2	21.3 (11.1)	30.8
radial strain	base	26.7 (25.6)	42.1	35.6 (29.6)	59.2
rotation (twist)	apex-base	5.7 (4.3)	4.0	12.3 (9.9)	59.2

Mean (SD) difference in mid-myocardium circumferential strain between visits was 6.7% (4.1), 6.5% (4.0) and 8.8 % (5.2) at apex, mid-LV and base respectively.

Mean (SD) difference in radial strain between operators was 28.1% (47.8), 17.6% (21.6) and 26.7% (25.6) and between visits was 7.3% (6.0), 21.3% (11.0) and 35.6% (29.6) at apex, mid-LV and base respectively.

The mean (SD) difference in mid-myocardium left ventricular twist between operators was 0.6^o^ (0.3^o^) or 5.7% (4.3) and between visits was 1.2^o^ (SD 0.9^o^) or 12.3% (9.9).

## Conclusions

The inter-observer and inter-study variability of circumferential strain and LV twist measured from CSPAMM tagged CMR data are low, but are higher for radial strain.

